# MiR-490-3p Silences CDK1 and Inhibits the Proliferation of Colon Cancer Through an LLPS-Dependent miRISC System

**DOI:** 10.3389/fmolb.2021.561678

**Published:** 2021-04-08

**Authors:** Da Qin, Rui Wei, Shengtao Zhu, Li Min, Shutian Zhang

**Affiliations:** Beijing Key Laboratory for Precancerous Lesion of Digestive Disease, Department of Gastroenterology, National Clinical Research Center for Digestive Disease, Beijing Digestive Disease Center, Beijing Friendship Hospital, Capital Medical University, Beijing, China

**Keywords:** MiR-490-3P, Cdk1, phase separation, proliferation, colon cancer

## Abstract

Liquid-liquid phase separation (LLPS) is a burgeoning concept in cell biology, which was associated with miRISC machinery. However, most studies about LLPS are based on overexpression of core proteins, which is far away from nature condition of cells, whether miRISC underwent LLPS under biological condition remains unknown. Taking miR-490-3p and its target CDK1 as an example, we revealed without overexpression of any protein components, miRISC functioned in an LLPS-depend manner. We firstly found miRISC has liquid-like properties in colon cancer (CC) cells and could fulfill common LLPS criteria under overexpression condition. Then, RIP was performed to confirm miR-490-3p is actually functioning in miRISC. RT-qPCR, western blot and luciferase assays were performed and found miR-490-3p could significantly decrease expression of CDK1 in both RNA and protein levels. However, without overexpression of miRISC components, when treating CC cells with 1,6-hexanediol(1,6-HD), a widely used LLPS inhibitor, the silence effects of miR-490-3p to CDK1 were totally abolished, no matter in RNA, protein or luciferase levels, suggesting that miRISC functions in an LLPS-depend way under biological condition. In conclusion, we found miR-490-3p could silence CDK1 to inhibit the proliferation of CC cells in an LLPS-depend manner.

## Background

Liquid-liquid phase separation theory proposed that biomolecular condensates form through liquid-liquid phase separation (Boeynaems et al., 2018), which is driven by interactions of multivalent molecules (Shin and Brangwynne, 2017). This emerging theory provides a theoretical basis for the formation of membrane-less organelles and cell compartmentalization. In 2019, a series of articles focused on LLPS published, highlight the participation of LLPS in many crucial biological processes, such as coactivators, paraspeckles, and stress granules (Patel et al., 2016; Du and Chen, 2018; Maharana et al., 2018; Muiznieks et al., 2018; Sabari et al., 2018). However, due to the high acute toxicity of 1,6-HD, the widely used LLPS blocker, there are still many problems unsolved in LLPS field.

MiRNA-induced gene silence is an important part of post-transcription regulation. Previous studies have revealed that many kinds of miRNAs play crucial roles in tumorigenesis. MiRNAs are proved to function in miRISC, which is a multi-protein assembly. Sheu-Gruttadauria and MacRae (2018) reported that Argonaute2 (Ago2) and TNRC6B, two protein components of this multi-protein complex, could condense into phase-separated droplets *in vitro*. However, their main conclusion was based on an overexpression system of Ago2 and TNRC6B. Considering proper protein concentration and pH level are crucial for the formation of phase-separated droplets (Boeynaems et al., 2018), whether miRISC has the ability to condense into phase-separated droplets without any overexpression of its main components is still unknown.

Taking miR-490-3p and its target gene CDK1 as an example, here we revealed that without overexpression of any components, miRISC still functioned in an LLPS-dependent manner, and LLPS directly determined the efficiency of miRISC.

## Methods

### Cell Culture and Transfection

RKO and HT-29 cells (purchased from the Shanghai Institute of Cell Biology, Shanghai, China) were cultured in DMEM medium. Cells were maintained at 37°C in a humidified atmosphere of 5% CO_2_, and transfection was performed using 5 μL of Lipofectamine 3000 with 2 μg of plasmids or 5 μL miRNA mimics.

### Co-localization, Fusing, and Fluorescence Redistribution After Photobleaching Experiments

Confirmational experiments were performed according to the common LLPS criteria under overexpression condition to prove its ability to LLPS. PmCherry-C1 plasmid containing AGO2 gene and pEGFP-C1 plasmid containing TRNC6B gene (Supplementary File 1) were purchased from YouBio, China. Cells transfected with both of these two plasmids were plated in cell culture dishes after transfection for 48 h. Laser scanning confocal microscopy (LSCM) was used for the observation of sub-cellular structures. To prove the fluidity of miRISCs, we firstly focused on the condensation shape of them. Secondly, we photographed the droplets every 1 min under LSCM to observe the movement of these complexes. Thirdly, FRAP was also performed to confirm the fluidity of miRISCs. After photobleaching the droplets for 4 s, we photographed the droplets every 1 min to monitor the fluorescence intensity changes of the bleached regions.

### 1,6-HD Concentration and Treatment Duration Detection

We performed a 1,6-HD concentration detection assay to find an appropriate concentration of 1,6-HD, which could inhibit the LLPS process without resulting in acute cell necrosis in a long-term treatment. Cells were plated in 6-well cell culture clusters. For quantitation of acute necrosis cells, 100 μL of propidium iodide (PI) solution was added to each well and all reacting system were incubated in incuCyte live cell system. Thus, red object integrated intensity could be a well measurement tool to assess acute necrosis cell numbers. Integrated intensity was collected every 20 min by using incuCyte live cell system.

### RNA-Immunoprecipitation (RIP) Assays

RNA-Immunoprecipitation (RIP) assay was then performed to directly confirm the colocalization between miR-490-3p and miRISC using Imprint^®^ RNA Immunoprecipitation Kit from Sigma, following its Anti Ago2-RNA Immunoprecipitation protocols. Cells were divided into 2 groups including IgG group (negative controls) and anti-Ago2 group. Cells from each group was 2 × 10^7^ to ensure enough cell amounts for RIP. After washing magnet breads, we pre-bind them to rabbit IgG antibody and anti-Ago2 antibody, separately. Then, we released cells from culture medium and added mild lysis buffer to lysis cells. We subsequently transferred cell lysate to resuspended beads and Incubate at 4°C with rotation O/N. After purifying RNA and reverse transcription following protocols from Takara, RT-qPCR was then performed to check expression level between IgG group and anti-Ago2 group. Stem-loop primer was applied to reverse transcription, and its sequence is “CTCAACTGGTGTCGTGGAGTCGGCAATTCAGTTGAGAG CATGA.” The PF primer sequence of miR-490-3p is “ACACTCCAGCTGGGCAACCTGGAGGACTC.” And the PR primer sequence is “TGGTGTCGTGGAGTCG.”

### Real Time-Quantitative PCR (RT-qPCR)

RT-qPCR was next performed to find out the effects of miR-490-3p to CDK1 and what would change if treated with 1,6-HD in RNA levels. In treated group, a total of 2 × 10^5^ cells were seeded in 6-well plates and/or transfected with 5 μL of miR-490-3p mimics by using Lipofectamine 3000 according to the manufacturer’s instructions in 1% 1,6-HD DMEM medium. In non-treated group, same cells were performed in normal DMEM medium, both miR-490-3p mimics and inhibitors were transfected separately by Lipofectamine 3000. Total RNA isolated from cells was obtained using TRIzol following the manufacturer’s protocol (Takara, Shiga, Japan). A total RNA (2 μg) was reverse-transcribed and cDNA was synthesized. The amplification of cDNA was performed in 12 μL reaction system following the Fast SYBR^TM^ Green Master Mix manufacturer’s instructions; GAPDH was used for normalization.

### Western Blot

Western blot assays were subsequently performed to check the effects of miR-490-3p to CDK1 and find out the change of these effects when treating with 1,6-HD. Cells were cultured and transfected with miR-490-3p mimics or normal controls in DMEM culture medium with 1% 1,6-HD or not for 48 h. We next lysed transfected cells in RIPA buffer and total protein was next obtained. After loaded protein onto an SDS-PAGE, we transferred protein to polyvinylidene fluoride (PVDF) membranes. After incubating PVDF membranes with 1:1,000 diluted CDK1-specific rabbit polyclonal antibody from Proteintech at 4°C overnight, then we washed them and incubated them with an anti-rabbit secondary antibody. Polyclonal antibodies of IGF1R, AKT/PKB, P38MAK, P44/42MAPK, and HER2 were also used to detect their expression levels under miR-490-3p overexpression condition. Finally, chemiluminescence (ECL) system (Millipore, Billerica, MA, United States) were used to visualize our results. Tubulin was used as endogenous reference protein.

### Luciferase Gene Reporter Assay

Luciferase assays were next performed to find out the binding position between miR-490-3p and CDK1 and observe the change when treated with 1,6-HD. PmirGLO plasmids containing CDK1 gene fragment (Supplementary File 2A) or mutant CDK1 gene fragment (Supplementary File 2B) were purchased from YouBio, China. A nature internal reference gene named hRluc-neo fusion gene was designed inside pmirGLO plasmid, which could equally express fluorescent protein as luc2 gene under non-disturbance conditions, to eliminate bias from transfection and other processes. In treat group, a total of 2 × 10^5^ cells were seeded in 6-well plates with 1% 1,6-HD DMEM medium and co-transfected with 2 μg of normal CDK1 luciferase reporter plasmids and/or 5 μL of miR-490-3p mimics by using Lipofectamine 3000 according to the manufacturer’s instructions. In normal group, same cells and treatment were performed as treat group except for treated with normal DMEM medium. In mutant group, same cells and treatment were performed as normal group except for transfected with 2 μg of mutant luciferase reporter plasmids. After the cells were cultured at 37°C for 36 h, luciferase activity was assessed using a Dual Luciferase Assay Kit (Thermo Fisher); all procedures were performed according to the protocol provided by Thermo Fisher. Luciferase activity values were measured and normalized to the corresponding hRluc-neo fusion gene activity values.

### MTS Assays

For the MTS assay, cells were plated in 96-well cell culture clusters (5,000 cells per well) after transfection with miR-490-3p mimics or normal controls for 24 h. For quantitation of the cell proliferation rate, 20 μL of 5 mg/mL MTS (thiazolyl blue) solution was added to each well and incubated for 2 h at 37°C. The absorbance was measured at 490 nm in a microplate spectrophotometer.

### Cell Cycle Assays

Cells were plated in 6-well cell culture clusters after transfected with miR-490-3p mimics or normal controls for 24 h. Then, cells were fixed with 70% ice-cold ethanol for at least 4 h. After incubating with 500 μL RNase (250 μg/mL) for 30 min and then 50 μg/mL PI at 4°C for 30 min avoiding light, flow cytometry were used for detection of cell cycle distribution.

### EdU Assays

For the EdU assay, cells were also plated in 96-well cell culture clusters (5,000 cells per well) after transfected with miR-490-3p mimics or normal controls for 12 h. For quantitation of the S-phase cells, 2 μL of EdU solution (ribobio, China) solution was added to each well and incubated for 2 h at 37°C. Then, after fixation following manufacturer’s instructions, we photographed cells in each wells and calculated S-phase cells proportion for further statistical analysis.

### Statistical Analysis

Statistical tests were performed using R 3.5.1^1^. Data are presented as the means ± standard deviations (*SD*s). Student’s independent t-test was used for statistical comparisons between the experimental and control groups. Every assay was performed at least triple times. *P* < 0.05 were considered statistically significant.

## Results

### IUPred2A Bioinformatic Analysis

**FIGURE 1:**
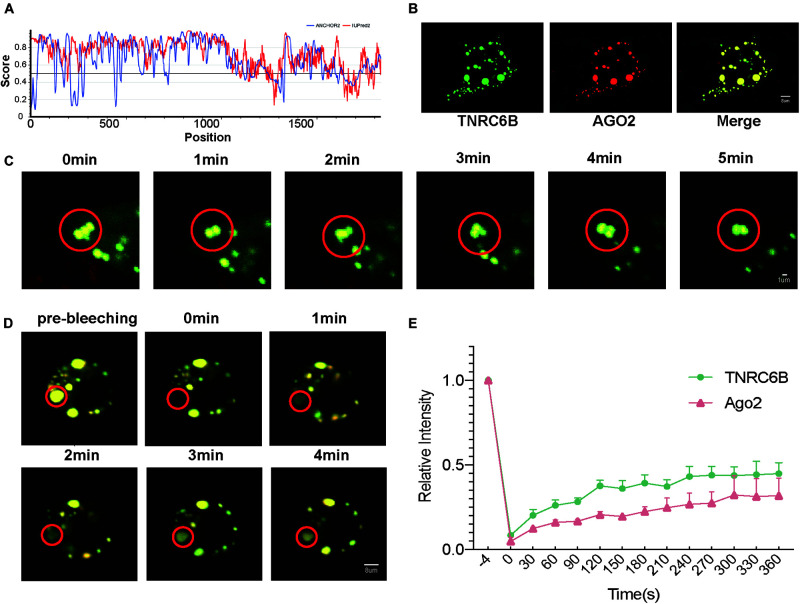
LLPS of miRISC in colon cancer cells under over-expression condition. **(A)** Bioinformatics analysis of the IDR of TNRC6B, two methods were performed to assess the IDR regions, red lines represent IUPred2 and blue lines represents ANCHOR2, regions above 0.5 represents IDR regions. **(B)** Co-localization of Ago2 and TNRC6B in RKO cells, and they could condense into large, spherical droplets. Green droplets represent TNRC6B and red droplets represents Ago2, yellow droplets represent the merge of both of 2 colors. **(C)** Droplets could move and fuse along with time elapse, yellow droplets represent the merge of both TNRC6B and Ago2. **(D)** Fluorescence intensity of Ago2 and TNRC6B recover slowly after photobleaching. Regions inside the red circle represent photobleaching regions. Yellow droplets mean the merge of both Ago2 and TNRC6B. **(E)** Fluorescence intensity of TNRC6B and Ago2 could recover after photobleaching along with time elapse, separately. Green line represents intensity of TNRC6B, red line represents intensity of Ago2.

At the beginning of this study, we firstly confirmed the potential of miRISC to LLPS. Intrinsic disordered regions (IDRs) are important to LLPS, proteins with IDRs were more possibly to induce LLPS. Researchers developed many bioinformatic methods to predict IDRs of proteins, IRPred2A is one of these important tools. In this study, IUPred2A (Mészáros et al., 2018) analysis showed that TNRC6B, a core protein of miRISC, has a long IDR (Figure 1A, regions above 0.5). This fact indicates that TNRC6B may play an important role in inducing LLPS.

### Co-localization, Fusing, and Fluorescence Redistribution After Photobleaching Experiments

According to the experimental guideline of LLPS (Boeynaems et al., 2018), common criteria for defining a phase-separated structure are that it is spherical, fuses, and could recover from photo-bleaching. Here we performed 3 assays as follows to prove LLPS in colon cancer cells. Firstly, after co-overexpression of Ago2 and TNRC6B, we observed that these two proteins well co-localized and condensed into large, spherical droplets which could represent miRISCs under LSCM in RKO cells (Figure 1B). Secondly, we observed that the spherical droplets could move and fuse with each other slowly (Figure 1C). Thirdly, the results of FRAP suggested that the fluorescence intensity of these two proteins could recover slowly after photobleaching (Figures 1D,E). Thus, the FRAP experiments gave us a stronger proof to confirm the fluidity of miRISCs under overexpression condition in colon cancer.

### 1,6-HD Concentration Detection Assays

**FIGURE 2:**
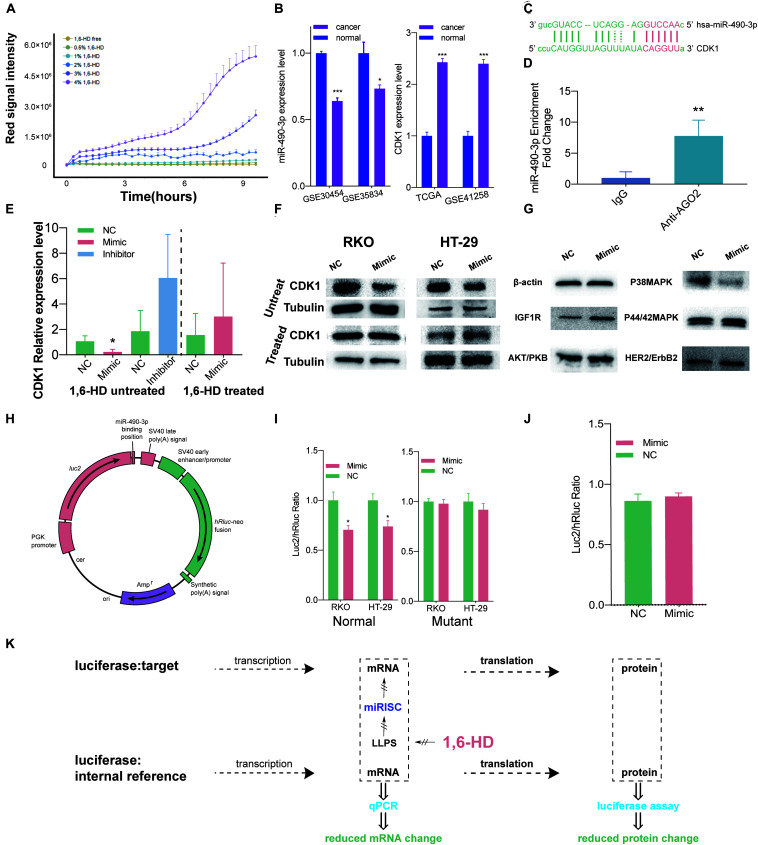
LLPS of miRISC in colon cancer cells under nature condition. **(A)** The relationship between 1,6-HD concentration and cell death. Red signal intensity represents necrosis cells number as time elapse. This figure illustrated the relationship between different 1,6-HD concentration and different treat time. 1% 1,6-HD treatment could hold less cell necrosis long with time elapse. **(B)** Expression level of miR-490-3p and CDK1 in TCGA and GEO database. Significantly lower expression level of miR-490-3p and higher CDK1 level were observed. **(C)** Predicted binding site between miR-490-3p and CDK1. **(D)** RIP analysis exhibits the colocalization between miR-490-3p and Ago2. **(E)** RT-qPCR assays under 1,6-HD treated and untreated condition. When not treated with 1,6-HD, lower CDK1 expression was observed in mimic group and higher expression was observed in inhibitor group. In treated group, this trend is disappeared. **(F)** Western blot shows less expression of CDK1 in mimic group when not treated with 1,6-HD and equally expression level in mimic group when treated with 1,6-HD. **(G)** Western blot shows less expression of P38MAPK in mimic group. **(H)** Profile of normal luciferase plasmid. **(I)** Luciferase assays of normal (left panel), mutant (right panel). Significantly lower CDK1 expression could be observed in mimic of normal group, which couldn’t be seen in mutant group. **(J)** Equal signal of luciferase assay could be observed when treated with 1,6-HD. **(K)** Ideograph for luciferase assays. *P < 0.05, **P < 0.01, ***P < 0.001.

After confirmation of miRISC’s LLPS property under overexpression condition, we started to explore whether LLPS could still be induced in miRISC under biological conditions. 1,6-HD is an important LLPS inhibitor, which could totally abolish LLPS. However, for long term treatment of cells, proper 1,6-HD concentration and duration is needed. In order to solve this problem, concentration and treat time detection assay was performed. The results from concentration detection assay suggested that the integrated intensity increased with the increase of 1,6-HD concentration. At 1% concentration, the integrated intensity was quite stable with long time treatment (Figure 2A). Thus, we choose 1% 1,6-HD to treat cells in the subsequent analysis.

### Differential Express Gene Analysis of Robust RNA Regulation Pair

Then, we started to take miR-490-3p and CDK1 as an example to explore the relationship between miRISC and LLPS under biological condition. Data from GEO (Balaguer et al., 2011; Pizzini et al., 2013; Martin et al., 2018) and TCGA (Muzny et al., 2012) suggested that colon cancer cells had lower miR-490-3p level and higher CDK1 level than normal colon mucosa cells (Figure 2B). Further TCGA analysis showed that miR-490-3p expression level was lower (Supplementary Files 3A,B) and CDK1 expression level (Supplementary Files 3C,D) was higher in every disease stage and in patients with or without lymph node metastasis. Figure 2C also showed a binding site between miR-490-3p and CDK1 predicted by Blast tools. These facts indicated that CDK1 could be a potential target for miR-490-3p.

### RIP Assay to Confirm the Colocalization Between miR-490-3p and miRISC

RIP assay was next performed to confirm that miR-490-3p function inside the miRISC. The result exhibits a greatly higher expression level in anti-Ago2 group, which was statistically significant (*P* = 0.0025 < 0.01, Figure 2D). This result gave us a strong evidence to show the colocalization between miR-490-3p and Ago2. Ago2 binds to TNRC6B as we have proved by live cell imaging technique (Figure 1B), which could be regarded as markers of miRISCs. All these facts confirmed that miR-490-3p colocalized with miRISCs.

### RT-qPCR and Western Blot Showed Abolished Silence Function of miRISC When Treated With 1,6-HD

RT-qPCR assays and western blot also provided clues for that miR-490-3p suppressed the expression of CDK1 and LLPS suppressor could abolish this effect in both RNA and protein levels.

RT-qPCR assays (Figure 2E) were next performed. We found that when treated with miRNA mimic, expression level of CDK1 was significantly lower, and it increased when treated with miRNA inhibitor at an α level of 0.1. However, when treated with 1,6-HD, miRNA mimics could no longer decrease CDK1 expression. Western blot was also performed to check CDK1 protein levels (Figure 2F). Our results showed that protein level of CDK1 decreased when cells were treated with miRNA mimics. However, after 1,6-HD treatment, miRNA mimic could no longer inhibit CDK1 expression, and this result is accordance with our RT-qPCR results. Also, we next detected other proliferation related genes including IGF1R, AKT/PKB, P38MAPK, P44/42MAPK and HER2, and found that protein level of P38MAPK decreased when cells were treated with miR-490-3p mimics (Figure 2G). Even though there are no putative targeting sites of miR-490-3p on the P38MAPK gene, it is also possible that miR-490-3p regulates P38MAPK in an indirect manner.

### Luciferase Assays Also Showed Abolished Silence Function of miRISC When Treated With 1,6-HD Disturbance

However, results of q-PCR and western blot were just providing clues for our findings, and there are still problems unsolved. Because of the complicated environment inside cells, unpredictable interferences could happen in no matter transcription, post-transcription or translation section when using a wide-spectrum suppressor like 1,6-HD. As a result, Estrogenic modified genes which could only regulated by miR-490-3p through miRISC are perfect tools to observe its function because it could be isolated observed. As a result, two plasmids were built for luciferase assays (Figure 2H). MiR-490-3p mimics resulted in a significant decreased luc2/hRluc ratio in colon cancer cells transfected with normal plasmid (Figure 2I, left panel), but not mutant plasmid (Figure 2I, right panel). These results indicated that miRISCs functioned normally and decreased luc2 expression by specifically interacting between miR-490-3p and predicted binding site (Figure 2C). However, when we treated RKO cells with 1% 1,6-HD, the difference in luc2/hRluc ratio between the two groups was eliminated (Figure 2J). Comparing to the results of Figure 2I, we can infer that miRISC has lost its function after treated with 1% 1,6-HD, a widely used LLPS inhibitor (Figure 2K).

### MTS, Cell Cycle Assays, and EdU Assays Indicated miR-490-3p Induce G1/S Arrest and Inhibit Cell Proliferation

**FIGURE 3:**
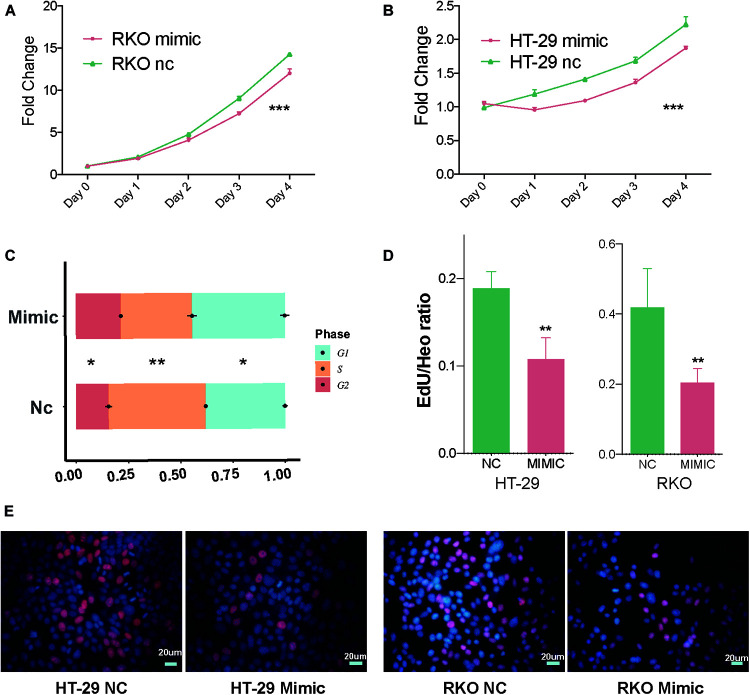
MiR-490-3p inhibits the proliferation of CC cells. **(A,B)** MTS assays of NC and mimic group showed significantly lower proliferation speed in mimic group. **(C)** Cell cycle assays showed miR-490-3p induced a significant G1/S arrest. **(D,E)** Edu assays between NC and mimic group showed miR-490-3p induced lower S phase cells. *P < 0.05, **P < 0.01, ***P < 0.001.

Additionally, after verifying miR-490-3p silences CDK1 through LLPS-dependent miRISC system, we further explored the biological function of miR-490-3p. MTS assays suggested that overexpression of miR-490-3p significantly decreased the proliferation of CC cells (Figures 3A,B). Cell cycle assays indicated that overexpression of miR-490-3p significantly induced G1/S arrest in CC cells (Figure 3C). Moreover, EdU assays revealed that overexpression of miR-490-3p significantly decreased the S-phase proportion of CC cells (Figures 3D,E). Thus, those results showed that miR-490-3p could inhibit the proliferation of CC cells.

## Discussion

Common criteria for defining a phase-separated structure are that it is spherical, fuses, and could recover from photo-bleaching (Alberti et al., 2019). However, such experiments were performed with over-expression of miRISCs core proteins, largely weaken the proof of LLPS in miRISCs. Previous studies have confirmed that the maintenance of phase separation state is closely related to environmental factors such as protein concentration and pH level. *In vitro* conditions, when sufficient protein concentration is provided, LLPS could be observed in many different proteins, even the ones not associated with LLPS process under natural condition. As a result, LLPS was not fully accepted by all of researchers. This study was designed to solve the problem whether LLPS happens or not under a non-overexpression condition.

Since there is no approach available to detect proteins directly under natural condition, we try to find an indirect way to confirm the LLPS process of miRISCs in CC cells under natural concentration of all key components of miRISC. It has been proved that the mRNA de-adenylation induced by miRISC could be accelerated greatly by LLPS *in vitro* (Sheu-Gruttadauria and MacRae, 2018). Thus, detecting the efficiency of miRISC machinery would be a possible alternative and substitute for LLPS.

1,6-HD is a widely used LLPS inhibitor, which can disrupt phase-separated subcellular structures by disrupting their multivalent hydrophobic interactions (Sabari et al., 2018). Most previous studies used high concentrated 1,6-HD for a short-term treatment to obtain acute biochemical change in LLPS. However, a long-term treatment is needed to observe changes in mRNA and protein level shifted by LLPS inhibition in miRISCs. Here we found that 1% 1,6-HD could inhibit the LLPS process without resulting acute cell death in a long-term treatment.

The choice of this RNA pair as our subject is because of its robustness. It is important for a new theory to find a stable platform to show its effectiveness. After careful bioinformatic analysis of 3 GEO database and TCGA database in colon cancer, we found that miR-490-3p was significantly down-regulated and CDK1 was significantly up-regulated. CDK1 is a functional protein closely related to cell cycle and easy to monitor its activity so that the effectiveness of our new theory could be well detected. Additionally, we used many bioinformatic tools to predict the target of miR-490-3p and found that CDK1 is a potential target for it. Taking all those together, we believed that miR-490-3p is a good model to study LLPS-based miRISC machinery.

Then, RT-qPCR and western blot were firstly performed to confirm the LLPS state of miRISC in cells without over-expression. However, a lot of disturbance caused by 1,6-HD happened in genomic, post-genomic, transcription levels. A minor reacting system which is more specific was needed. As a result, luciferase assays were performed to further prove our theory. Predicted binding site (Figure 2C) was inserted in MCS region lying in 3′-UTR of luc2 gene inside our luciferase reporter plasmid. The expression of luc2 gene and hRluc gene will change proportional except for the inserted sequence is specifically affected. When we treated cells with 1% 1,6-HD, the difference in luc2/hRluc ratio between the two groups was eliminated (Figure 2J). Comparing to the results of Figure 2I, we can infer that miRISC has lost its function after treated with 1% 1,6-HD (Figure 2K). However, our study still has some limitations. Only miR-490-3p was used for validation of our results, more subjects would help to improve its generality. We are still focusing on more miRNAs or other molecules underwent LLPS to generalize our theory, which will be published in the near future. Furthermore, we are also focusing on new method and more specific inhibitors of LLPS to make a difference in this filed. Thirdly, our results are mainly based on experiments in cell line levels. As a novel hypothesis, the tools in LLPS studies are too simple and crude which could not support researches on animal or human tissues. We will confirm our findings in animal or human tissue levels when such technique approaches are ready. Also, we will try our best to find out proper experiment methods to confirm LLPS in animals or human tissues levels.

Given plenty of substrates (target mRNA) and identifiers (miRNA), miRISC could have functioned heavily to degrade its targets (Figure 2K). With a treatment of low dose LLPS suppressor, the miRISC machinery was totally inactivated, suggested that miRISC functions in an LLPS-depend way under its nature condition, which hasn’t been reported in previous studies. Here we also provided a pipeline combining low dose 1,6-HD treatment and miRISC efficiency detection to monitor the change of LLPS process without overexpression key LLPS components.

After verifying the silencing effects of miR-490-3p on CDK1 through an LLPS-dependent miRISC system, we further explored subsequent cellular effects. A series of assays indicated that miR-490-3p induced G1/S arrest and inhibited cell proliferation. These findings were in accordance with the change of CDK1 level, which suggested that miR-490-3p might inhibit the proliferation of CC cells through silencing CDK1. Even though CDK1 is one of the main regulators in cell cycle and proliferation, the causality between miR-490-3p-CDK1 and miR-490-3p-proliferation was not solid, and need further investigation.

## Data Availability Statement

The original contributions presented in the study are included in the article/Supplementary Material, further inquiries can be directed to the corresponding author/s.

## Author Contributions

DQ, LM, and SZha conceived and designed the study. DQ and RW performed all experiments. RW and SZhu helped to collect, reformat, and analyze the primary data. DQ and LM drafted the manuscript. SZha proofread and revised the manuscript. All authors approved the final version of the manuscript.

## Conflict of Interest

The authors declare that the research was conducted in the absence of any commercial or financial relationships that could be construed as a potential conflict of interest.

## Footnotes

1 www.r-project.org

## Abbreviations

1,6-HD: 1,6-hexanediol
Ago2: Argonaute2
CC: colon cancer
FRAP: fluorescence redistribution after photobleaching experiments
IDR: internal disordered region
LLPS: liquid-liquid phase separation
LSCM: laser scanning confocal microscopy
miRISC: miRNA-induced gene silencing complex
PI: propidium iodideI
qPCR: real-time quantitative polymerase chain reaction.
