# Supplementary figures and images for: MiR-490-3p Silences CDK1 and Inhibits the Proliferation of Colon Cancer Through an LLPS-Dependent miRISC System
Source: Front Mol Biosci. 2021 Apr 8;8:561678. doi: 10.3389/fmolb.2021.561678 (PMC8060497; doi:10.3389/fmolb.2021.561678)

Project: YA3166F ABX2380-2 CDK1.SQD Contig 28

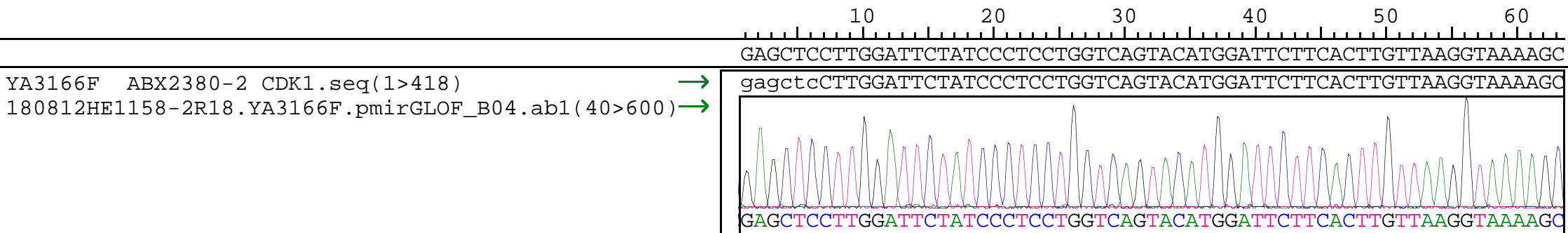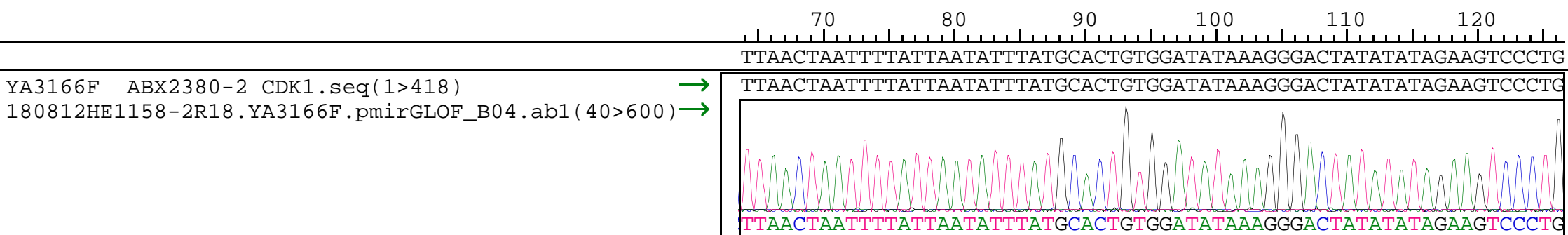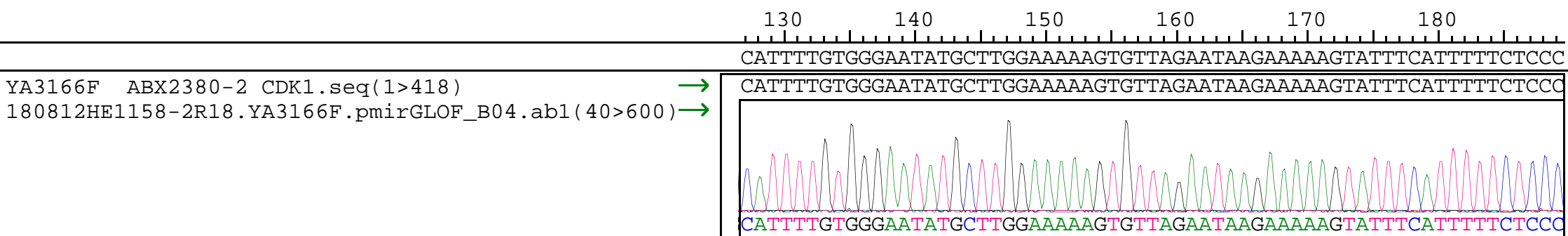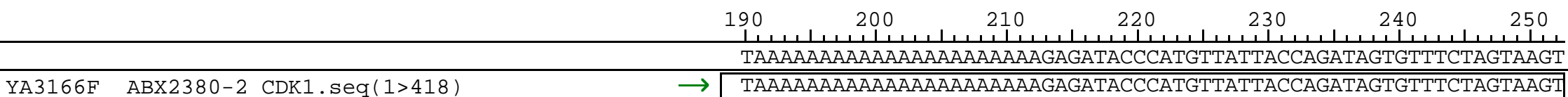

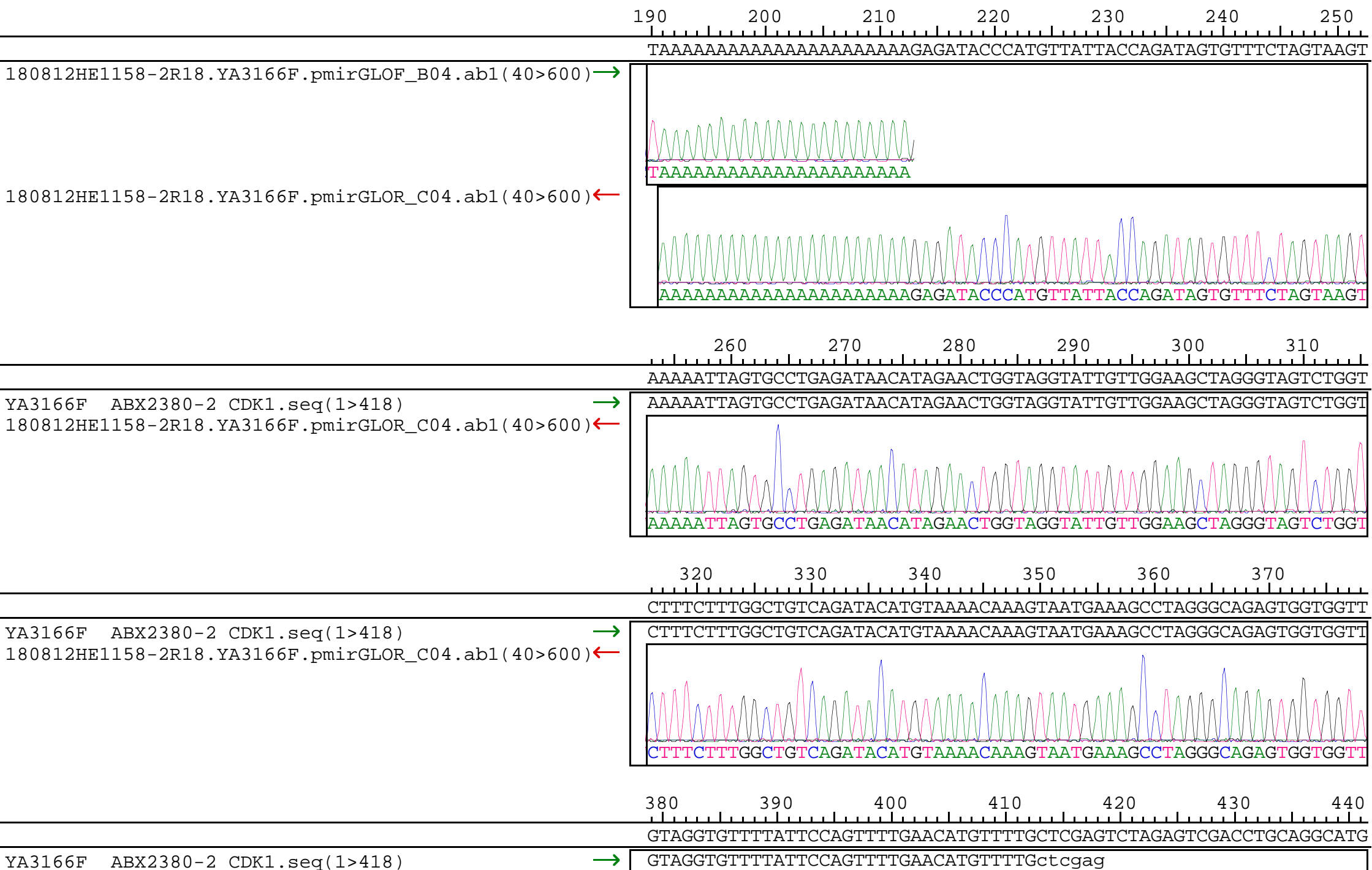

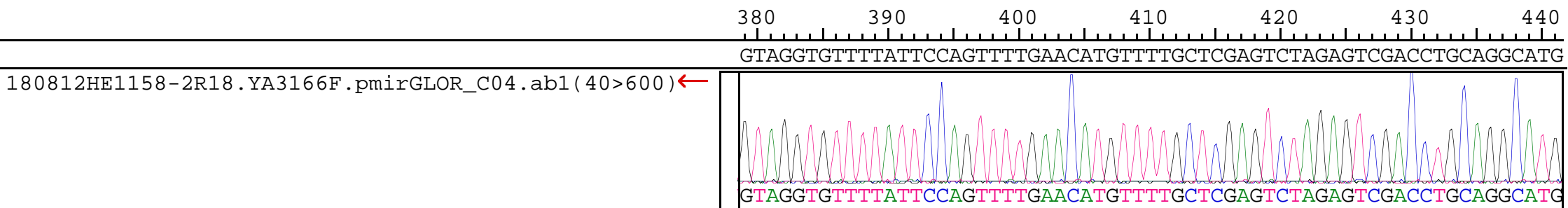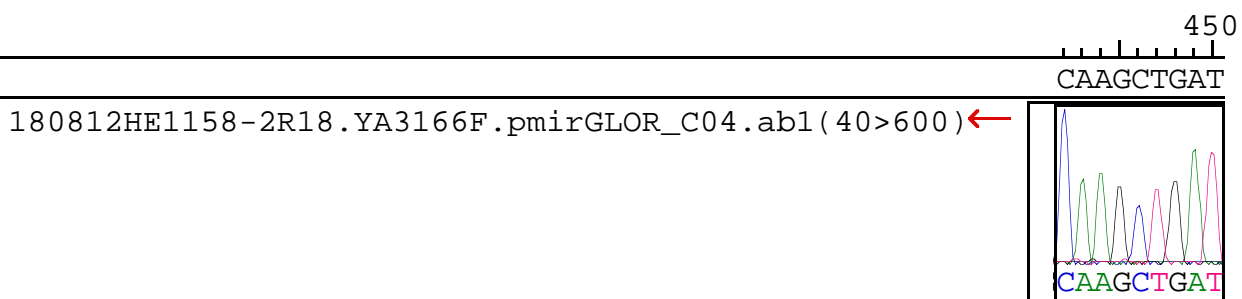

Supplement: Supplementary file 1 [file Data_Sheet_1.PDF]

Project: YA3165F ABX2380-1 CDK1.SQD Contig 45

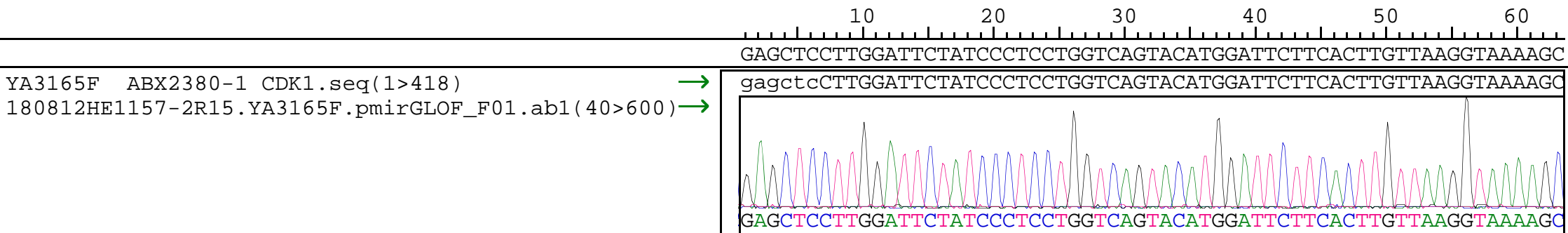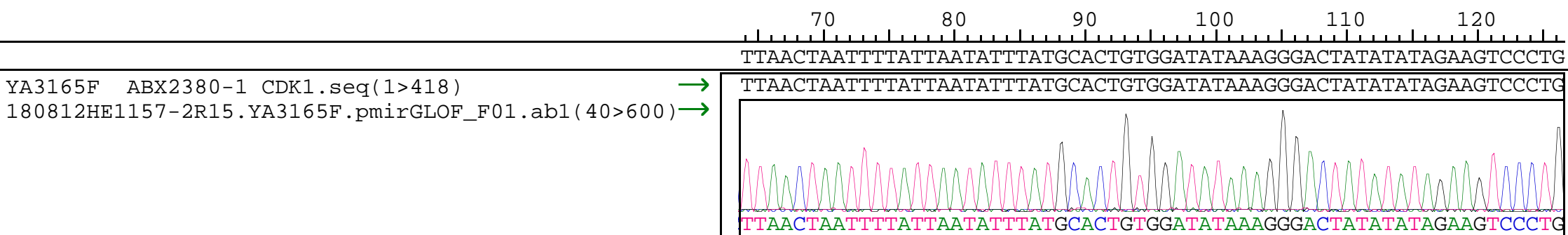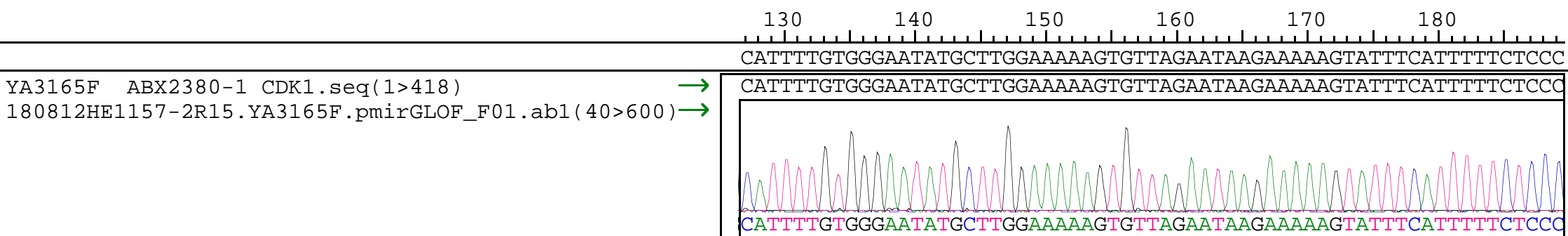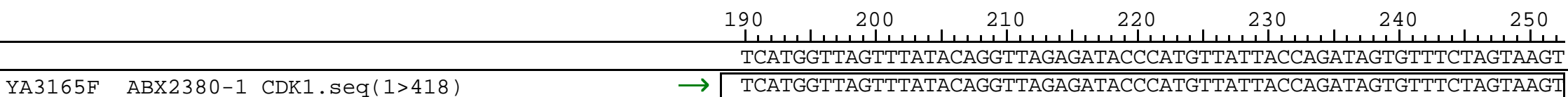

Project: YA3165F ABX2380-1 CDK1.SQD Contig 45

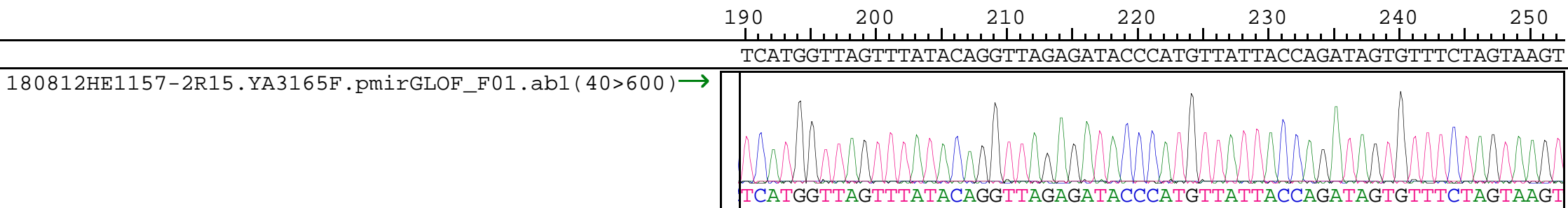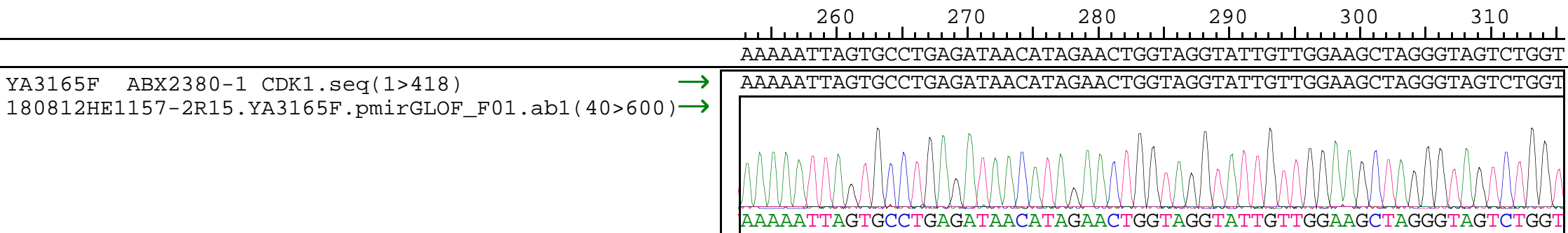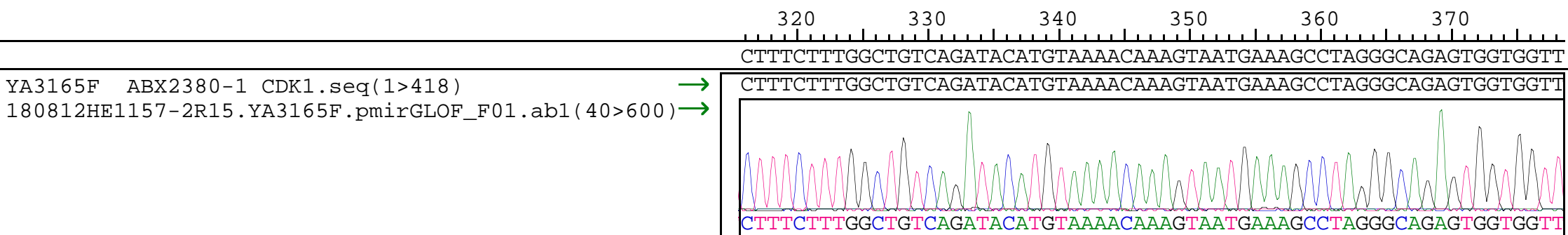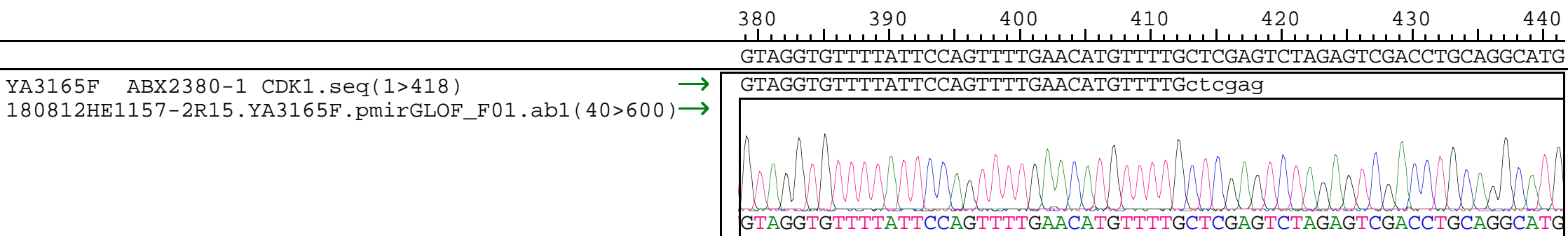

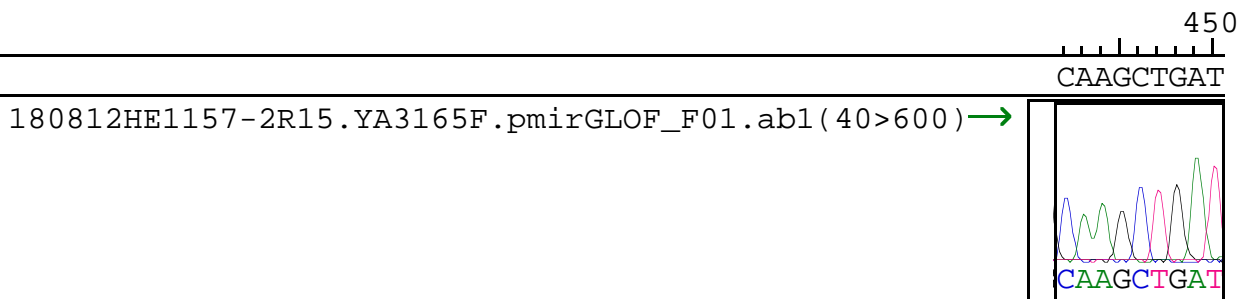

Supplement: Supplementary file 2 [file Data_Sheet_2.PDF]

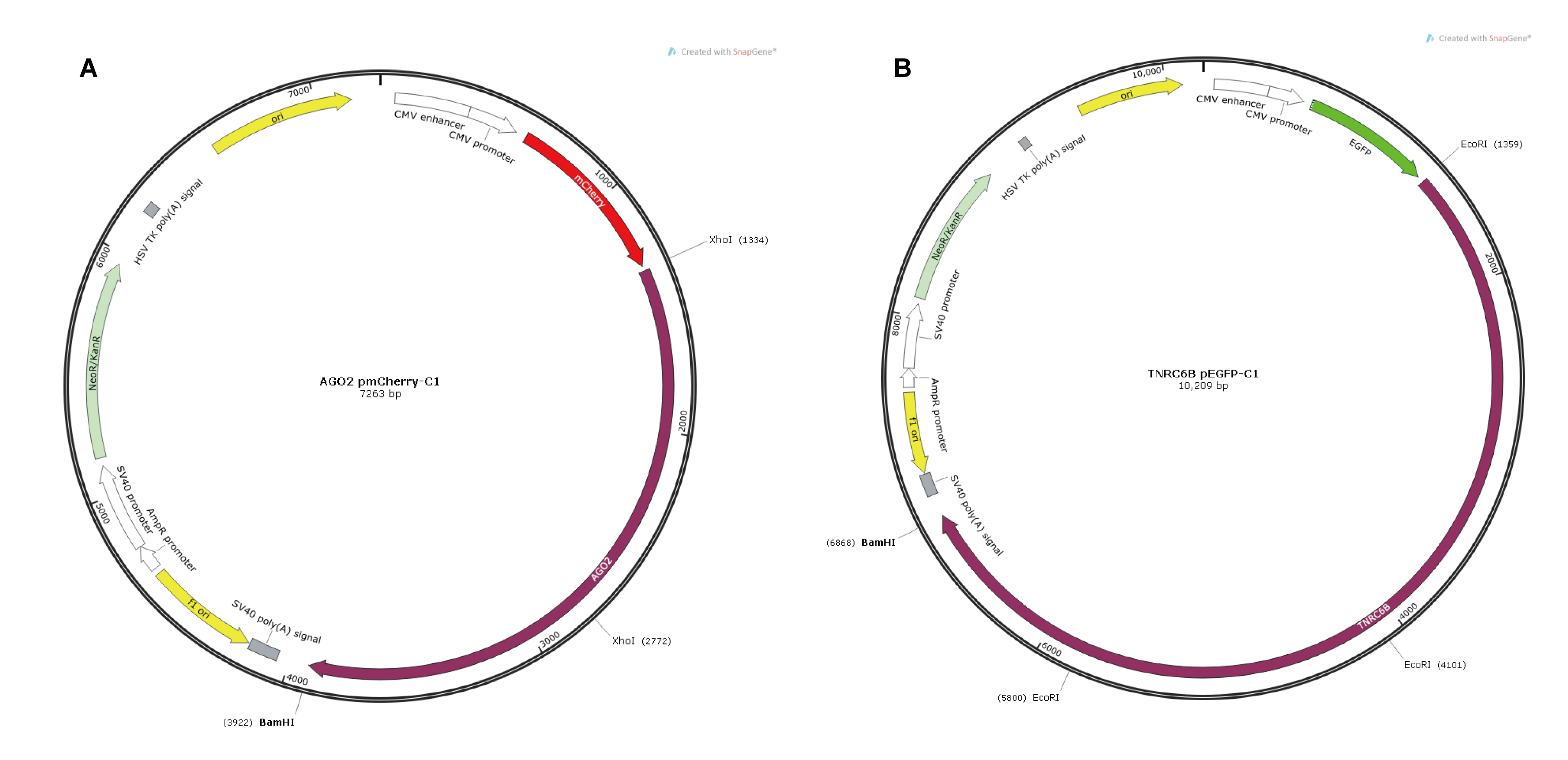

Supplement: Supplementary file 3 [file Image_1.PNG]

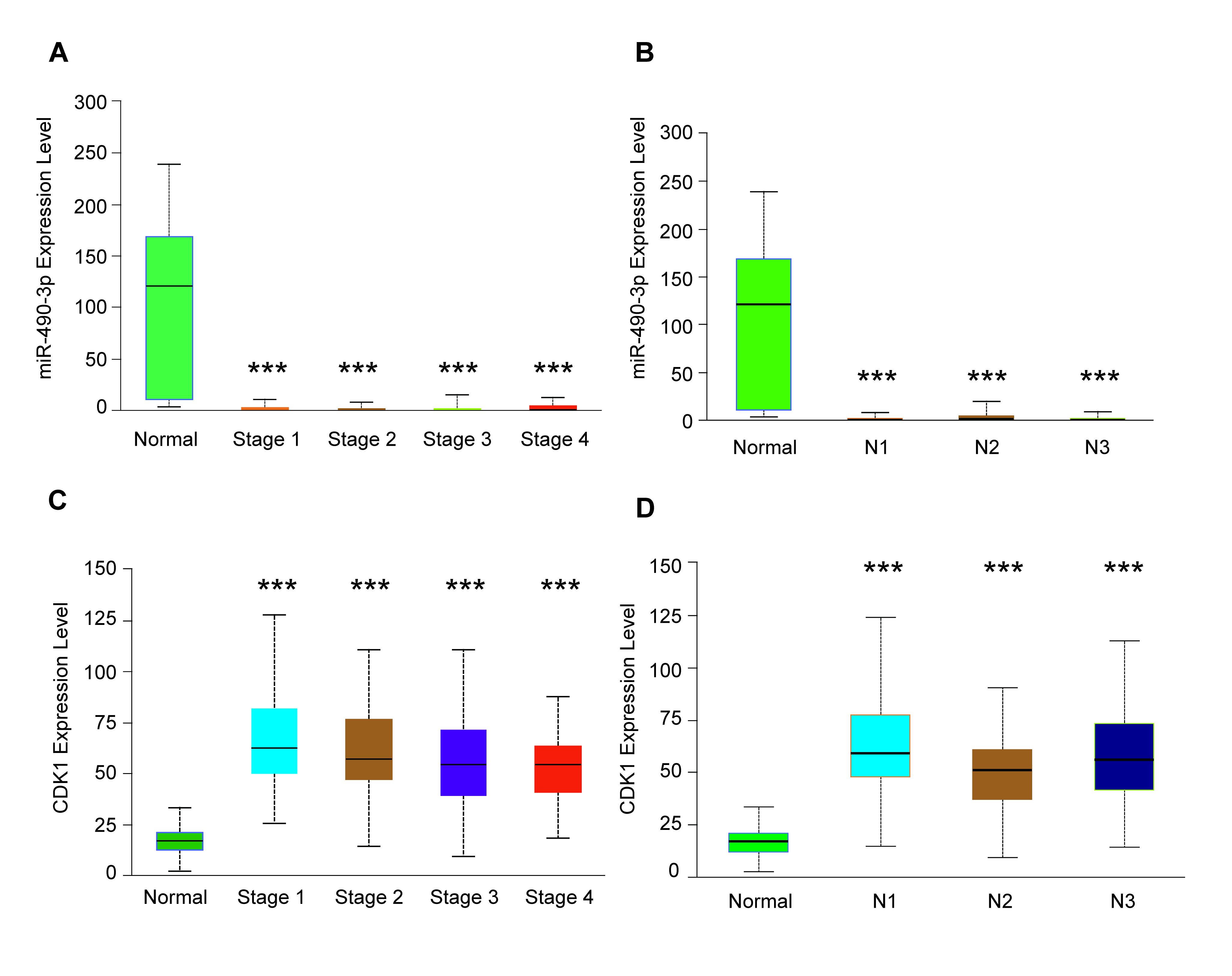

Supplement: Supplementary file 4 [file Image_2.TIF]
